# Visual prosody in Korean Sign Language: (non)manual cues for boundary and prominence

**DOI:** 10.3389/fpsyg.2025.1601842

**Published:** 2025-10-21

**Authors:** Jungah Lee, Youngju Choi

**Affiliations:** 1 Chungbuk National University, Department of General Education, Chungju, Republic of Korea; 2 Chosun University, English Language and Literature Department & Department of Applied Linguistics and Sign Languages, Gwangju, Republic of Korea

**Keywords:** Korean Sign Language (KSL), sign language prosody, visual prosody, nonmanual cues, manual cues, prominence, prosodic boundary, accentual phrase (AP)

## Abstract

**Introduction:**

This study examines how manual and nonmanual features contribute to prosodic marking in Korean Sign Language (KSL), particularly for prominence and Accentual Phrase (AP) boundaries. While previous studies have emphasized the role of nonmanuals in marking prosodic boundaries, we investigate whether these cues in KSL primarily serve to indicate prominence, regardless of boundary position.

**Methods:**

Six adult Deaf KSL signers participated in a controlled card-arrangement task designed to elicit target signs in four prosodic conditions: focused vs. unfocused prominence and AP-initial vs. AP-medial positions. The resulting data were analyzed using Bayesian mixed-effects modeling, with two predictors: prominence (focused vs. unfocused) and boundary position (AP-initial vs. AP-medial). A range of manual and nonmanual features—including eye contact, eyebrow movements, and sign duration—were annotated and statistically evaluated to determine their association with prosodic prominence and boundary marking in KSL.

**Results:**

The results showed that prominence had a robust effect on both manual and nonmanual cues. Features like eye contact, furrowed eyebrows, and squinted eyes were significantly more frequent in focused conditions. In contrast, boundary position alone showed minimal impact, with few features differing between AP-initial and AP-medial positions. Although some interaction effects were found, they were not consistent across features.

**Discussion:**

These findings suggest that KSL prosody is prominence-driven, with nonmanuals functioning as primary markers of focus rather than of AP boundaries. By highlighting the prominence-driven nature of prosodic marking in KSL, this study contributes to a growing body of cross-linguistic research showing that prosodic strategies in sign languages are not uniform but shaped by language-specific implementations.

## 1 Introduction

Spoken languages convey grammatical and prosodic information through both segmental and suprasegmental cues. Grammatical markers—such as verb inflections in English and sentence-final suffixes in Korean—and prosodic features like intonation, stress, and rhythm collaboratively serve to organize syntactic structures, distinguish sentence types, and highlight prominence (Cho, 2016; Jun, 2006, 2010).

Sign languages similarly encode grammatical and prosodic structure using both manual articulators (e.g., timing, repetition, size, and location of signs) and nonmanual signals (e.g., facial expressions, head movements, eye gaze, and mouth gestures; Meier, 1993; Brentari, 2010; Sandler and Lillo-Martin, 2006; Stokoe, 2005; Nespor and Sandler, 1999). Among these, nonmanual features play a particularly salient role in expressing prosodic prominence—functions typically realized by pitch accent and stress in spoken languages (Sandler, 2010; Dachkovsky et al., 2013). Raised eyebrows, widened eyes, head movements, and directed gaze have been identified as key markers of informational focus in many sign languages (Wilbur, 2000, 2013; Crasborn and Van der Kooij, 2013; Fenlon and Brentari 2021). These cues frequently co-occur in coordinated clusters, generating visually salient markers of prominence. For example, in Sign Language of the Netherlands (NGT) and Italian Sign Language (LIS), prominence is expressed through eyebrow movements, intensified mouthing, and head tilts (Crasborn and Van der Kooij, 2013; Geraci, 2015; Branchini and Mantovan, 2020; Fontana and Caligiore, 2021; Sbranna et al., 2023).

In spoken languages, prominence is typically realized through prosodic modulation of pitch, stress, and duration to signal focus (Beckman and Pierrehumbert, 1986; Crystal, 2011). These modulations enhance the perceptual salience of target elements and often influence the articulation of adjacent segments—a phenomenon known as prosodic strengthening (Cho, 2016; Mücke and Grice, 2014). In Korean, prominence commonly appears on focused elements within a prosodic phrase and is phonetically marked by a combination of pitch rise, increased amplitude, and temporal expansion, particularly through pre-boundary lengthening (Cho, 2016; Jun, 2006). These phonetic cues are closely tied to prosodic structuring: Focus may trigger prosodic restructuring, resulting in the formation of a new Accentual Phrase (AP; Jun, 2006), or it may be realized within an existing AP through localized phonetic enhancement in AP-medial positions (Cho, 2022). This distinction has generated ongoing theoretical debate about the relationship between prominence and prosodic phrasing.

In Korean Sign Language (KSL), however, the prosodic realization of prominence—especially through nonmanual articulations—remains underexplored. Prior studies have largely focused on syntactic strategies such as topicalization and focalization (National Institute of Korean Language, 2021), while empirical investigations of how prominence is marked prosodically, particularly at levels below the Intonational Phrase (IP), are limited. Although nonmanual features such as head nods and eye gaze have been observed at IP boundaries—paralleling patterns in other sign languages—it remains unclear whether these cues also function to mark focused elements within smaller prosodic domains like the AP.

Of particular interest is the question of whether KSL signers use (non)manual cues—such as eyebrow movement and/or head orientation—to mark prominence at the level of the AP. This raises broader theoretical questions about the interaction between boundary and prominence: Does prominence in KSL trigger the formation of a new AP, as in Jun’s (2006) account of spoken Korean, or can prominence be expressed within an existing AP without boundary modification, as suggested by Cho (2022)? Understanding how prominence is encoded at the AP level in KSL not only contributes to our knowledge of sign language prosody but also informs cross-modal comparisons of prosodic systems.

To address these questions, the present study investigates the use of (non)manual features in marking prominence and prosodic boundaries in KSL, focusing specifically on AP boundaries. We analyze several nonmanual cues—eye contact, furrowed eyebrows, squinted eyes, wide eyes, mouthing, etc.—under controlled conditions manipulating both prominence (focused vs. unfocused) and boundary position (AP-initial vs. AP-medial).

This study is guided by the following research questions:

(1) How do KSL signers use (non)manual features to mark prominence and prosodic boundaries in natural signing? Specifically, how are these features influenced by prominence condition (focused vs. unfocused) and boundary position (AP-initial vs. AP-medial)?
(2) To what extent do specific nonmanual features preferentially signal either prominence or boundary? Are certain features more frequently associated with focused elements regardless of position?
(3) How do KSL prominence-marking patterns compare to those in spoken Korean and other sign languages? Do similar mechanisms of prosodic enhancement and structuring appear cross-modally?

By situating KSL within broader typological and theoretical discussions of prosody, this study contributes to a deeper understanding of how modality shapes the interplay between prominence and prosodic structure.

## 2 Materials and methods

### 2.1 Participants

Six female Deaf signers (aged 40–58) participated in the study. Recruited through personal networks, they were compensated for their time (see Table 1 for demographic information)^1^. Participants completed a background survey and took part in a card arrangement game designed to elicit naturalistic signing and to examine how nonmanual cues mark prominence and AP boundaries in KSL^2^. A KSL interpreter with over 15 years of experience and the second author ensured a comfortable environment. The experimenter, familiar with the KSL community, emphasized voluntary participation and allowed participants to withdraw at any time. Before recording, participants reviewed and signed consent forms (IRB: 2-1041055-AB-N-01-2024-24). All participants had completed higher education and maintained strong connections with Korea’s Deaf community.

**Table 1:** Demographic information of participants.

Demographic information	Description
Mean age	47.8 Y (SD: 7.2)
Hometown	Chonnam (west southern province)
Mean age of first exposure to KSL	10.2 Y (SD: 4.3)
Mean length of using KSL	37.5 Y (10.4)
Education background	College
Occupation	Professional office worker

### 2.2 Materials and task design

The target signs consisted of four minimal pairs in KSL—^3^chicken vs. silly, see vs. find, practice vs. non-deaf, and pretty vs. difficult—differing in one phonological parameter (location, movement, orientation, or handshape). These pairs were chosen to ensure clear phonological contrasts, as minimal pairs inherently differ by only one phonological parameter. This parametric difference allows for precise control of segmental variation, making focus types (focused vs. unfocused) and prosodic conditions (AP-initial vs. AP-medial) more salient and analytically tractable. Supplementary material summarizes the target signs.

To elicit natural prosodic conditions, we employed a card arrangement task inspired by Choi et al. (2020). Participants were asked to respond to spatial prompts by arranging two types of cards: one set depicting lexical signs such as silly and chicken, and another set displaying images of objects such as trees and flowers. This task was designed to generate discourse contexts in which prosodic marking of prominence and phrasal boundaries could naturally emerge. A key observation from this task was the consistent production of a prosodic pause following the negation sign no. In participants’ responses, no reliably marked the end of the preceding prosodic unit and was immediately followed by the onset of a new prosodic phrase. This boundary was perceptually confirmed by both a professional KSL interpreter and the second author, a sign language specialist.

**Figure 1:**
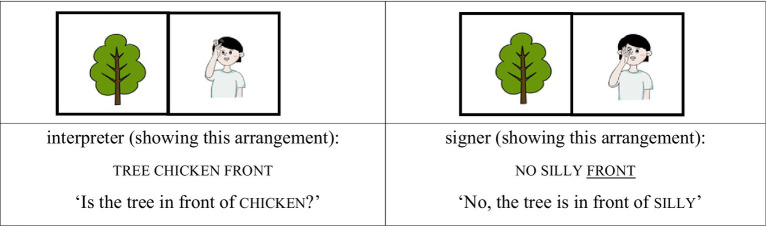
Card arrangement example for AP-initial focused condition.

**Figure 2:**
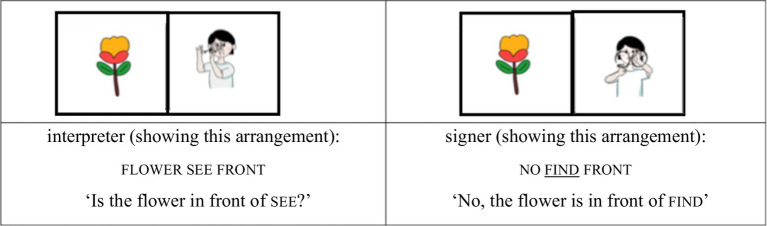
Card arrangement example for AP-initial focused condition.

We treated the lexical sign that immediately followed no—and thus occurred after a prosodic reset—as occupying the initial position of a new accentual phrase (AP-initial position). For instance, when the interpreter placed the card depicting the sign silly in front of the card with a tree image and asked, “Is the tree in front of chicken?,” the signer responded, “No, the tree is in front of silly.” In this response, silly follows a clear pause after no, marking the beginning of a new prosodic phrase. Because silly is the first lexical item in the phrase and also carries contrastive focus, this condition is classified as AP-initial focused (see Figures 1, 2).

(1) AP-initial focused condition

**Figure 3:**
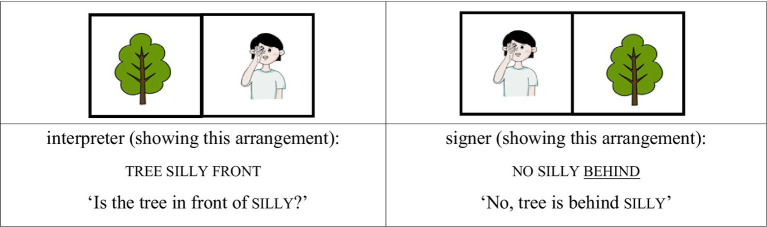
Card arrangement example for AP-initial unfocused condition.

**Figure 4:**
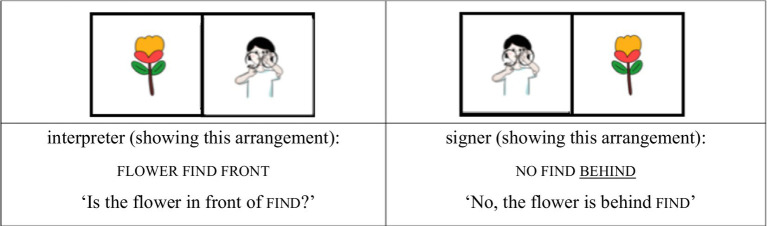
Card arrangement example for AP-initial unfocused condition.

For the AP-initial unfocused condition, the same structure was used, but contrastive focus was shifted away from the noun. For example, when the tree was placed behind silly, the signer responded, “No, the tree is behind silly,” with the focus on the locative expression behind rather than on the noun silly. Although silly still occupies the AP-initial position, it is not the focused element (see Figures 3, 4).

(2) AP-initial unfocused condition

To construct the AP-medial conditions, we visually modified the target card by overlaying a color (e.g., purple) on the background of the silly card. This created a modified sign such as purple silly, where the adjective purple appears first and the noun silly follows. Importantly, this manipulation ensured that silly no longer occurred in the AP-initial position but rather in the AP-medial position, following the color modifier.

**Figure 5:**
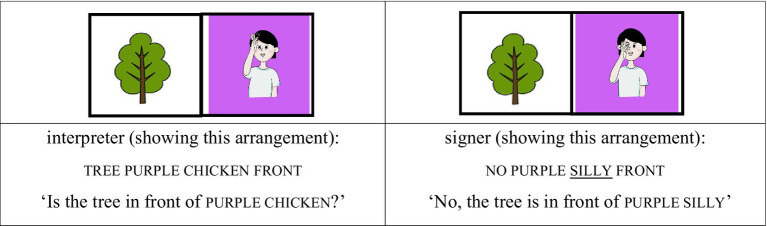
Card arrangement example for AP-medial focused condition.

**Figure 6:**
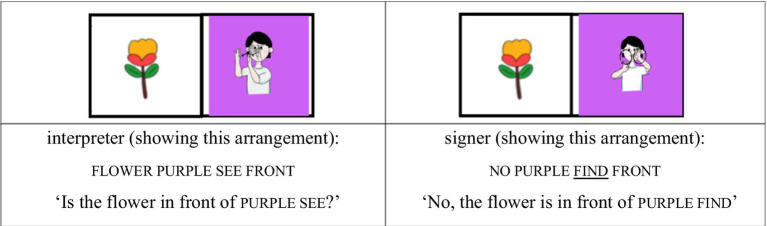
Card arrangement example for AP-medial focused condition.

In the AP-medial focused condition, for instance, the interpreter placed the purple silly card in front of the tree card and asked, “Is the tree in front of the purple chicken?” The signer responded, “No, the tree is in front of the purple silly,” shifting contrastive focus from chicken to silly. Because silly appears after the AP-initial modifier purple, it is prosodically situated in the AP-medial position, even though it bears informational focus (see Figures 5, 6).

(3) AP-medial focused condition

**Figure 7:**
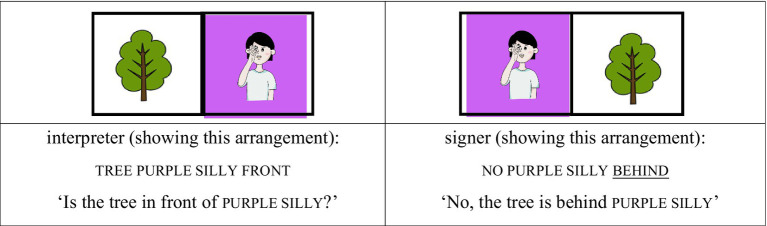
Card arrangement example for AP-medial unfocused condition.

**Figure 8:**
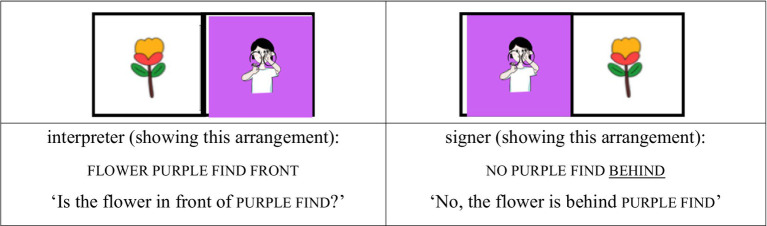
Card arrangement example for AP-medial unfocused condition.

For the AP-medial unfocused condition, the same card design was used, but the focus was shifted to another element. When the purple silly card was placed behind the tree and the interpreter asked, “Is the tree in front of the purple silly?,” the signer replied, “No, the tree is behind the purple silly,” assigning contrastive focus to behind rather than to the noun phrase. In this case, silly remains in the AP-medial position without bearing prosodic prominence (see Figures 7, 8).

(4) AP-medial unfocused condition

The card arrangement game was conducted in KSL without pre-scripted sentences or predetermined structures. The provided examples illustrate the study’s design and target prosodic conditions but do not constitute fixed experimental prompts (Choi et al., 2020). The KSL interpreter and the signers interacted freely to ensure authentic prosodic phrasing and spontaneous responses.

In summary, the determination of AP-initial and AP-medial positions was based on two key observations consistently confirmed across participants: (1) the presence of a prosodic pause immediately following no; and (2) the grouping of purple and the following target sign into a single Accentual Phrase (AP). These prosodic structures were independently confirmed by a professional KSL interpreter and the second author, a sign language specialist.

### 2.3 Recording procedure and analyses

**Figure 9:**
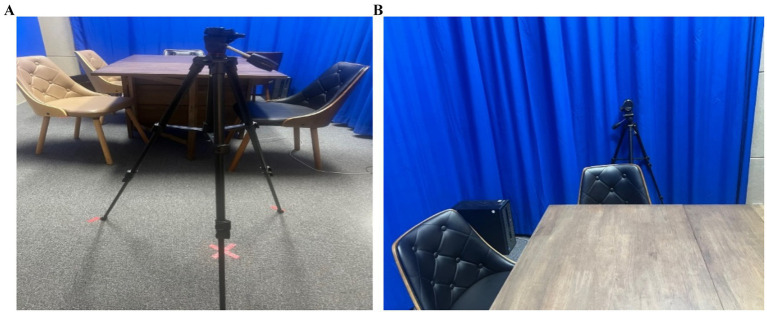
**(A)** (Left) and **(B)** (Right). Camera setting for consistent video recording process.

Before recording, participants completed a 30-min practice session to familiarize themselves with the target word cards and the card arrangement task. They reviewed the game structure and confirmed their understanding of the rules. Recording took place in a private, controlled setting at C University, with only the KSL interpreter and the signer present to ensure natural interaction. Two high-definition cameras (Panasonic HC-VX1) were positioned to clearly capture the interpreter, the signer, and their upper-body movements (see Figures 9A,B). The card arrangement game lasted about an hour, with standardized lighting and room configurations to ensure consistency.

Each test set was repeated three times in a randomized order, resulting in 971 observations (6 signers × 8 target signs × 2 boundary positions × 2 focus types × 3 repetitions). Of the 971 tokens, 967 lexical sign tokens were analyzed. This number excludes a small set of tokens due to unclear articulation, annotation uncertainty, or occasional omissions by the interpreter during simultaneous translation. These exclusions were necessary to ensure the reliability of prosodic coding and acoustic analysis. Table 2 presents the frequency of tokens across boundary position and prominence condition. A trained KSL interpreter transcribed and glossed the nonmanual features following established conventions (see Table 3; FACS and coding systems in KSL; Ekman and Friesen, 1978; Cohn et al., 2007; Dachkovsky and Sandler, 2009, Dachkovsky et al., 2013; National Institute of Korean Language, 2021). The interpreter, with over 15 years of experience working with KSL, collaborated with the second author to apply a structured coding protocol. To ensure both accuracy and consistency, the first author and the corresponding author conducted a final review of the transcriptions for quality assurance. The annotated dataset is provided as supplementary material (OSF repository).^4^,^5^

**Table 2:** The number of tokens across boundary and prominence condition.

Boundary	Prominence	The number of tokens (frequency)
AP-initial position	Focused	281
	Unfocused	206
AP-medial position	Focused	269
	Unfocused	211
Total		967

**Table 3:** Glossing & coding of (non)manual features.

Nonmanual features					Manual features				
Eyebrows	Eyes	Mouth	Head	Upper body	Space	Speed	Pause	Hold	Intensity
raise	squint	mouth gesture	head forward	narrow shoulder	small	slow	short	short	soft
grimace	wide open	mouthing	head nod	lowering shoulders	big	fast	long	long	intense
lowering	contact (intense gaze)	lip rounding	head up	bending the upper body					
		pucker	head down	lean back					
		tight lips	lean left	lean left or right					
		upper lip raise	lean right						

Prosodic boundaries in this study were identified using a data-driven approach based on observable (non)manual cues in the signers’ productions, including pauses, final-sign lengthening, holds, and the initiation of nonmanual signals. Importantly, the identification of prosodic boundaries was driven by perceptual and visual evidence emerging from signers’ actual productions, rather than by syntactic structure. This approach ensured that prosodic segmentation reflected naturally occurring signing patterns. By integrating general prosodic theory with KSL-specific discourse organization, our approach ensures theoretical rigor while remaining sensitive to the modality-specific nature of sign language prosody.

## 3 Results

To investigate the effects of prominence (focused vs. unfocused) and boundary (AP-initial vs. AP-medial) on the frequency of (non)manual features, we conducted statistical analyses using R version 4.0.2 (R Core Team, 2019) with the tidyverse package version 1.3.0 (Wickham et al., 2019). Effect sizes (Cohen’s *d*) were computed using the effsize package version 0.8.0 (Torchiano, 2020). Bayesian mixed-effects regression models were implemented with brms (version 2.13.3; Bürkner, 2017). The Bayesian mixed models were specified with (Non)manual Features as the dependent variable and boundary (AP-initial vs. AP-medial) and prominence (focused vs. unfocused) as fixed effects, along with their interaction. Random effects included by-Signer intercepts to account for individual variability. Models were run using four Markov chain Monte Carlo (MCMC) chains with 6,000 iterations per chain, including 3,000 warm-up iterations, yielding a total of 12,000 post-warmup draws. Convergence diagnostics were assessed using *R̂* values, with all parameters reaching *R̂* ≈ 1.00, indicating proper mixing. Effective sample sizes (Bulk_ESS and Tail_ESS) were also examined to confirm model reliability.

(1) (Non)manual Features ~ Boundary*Prominence + (1|Signer)

**Figure 10:**
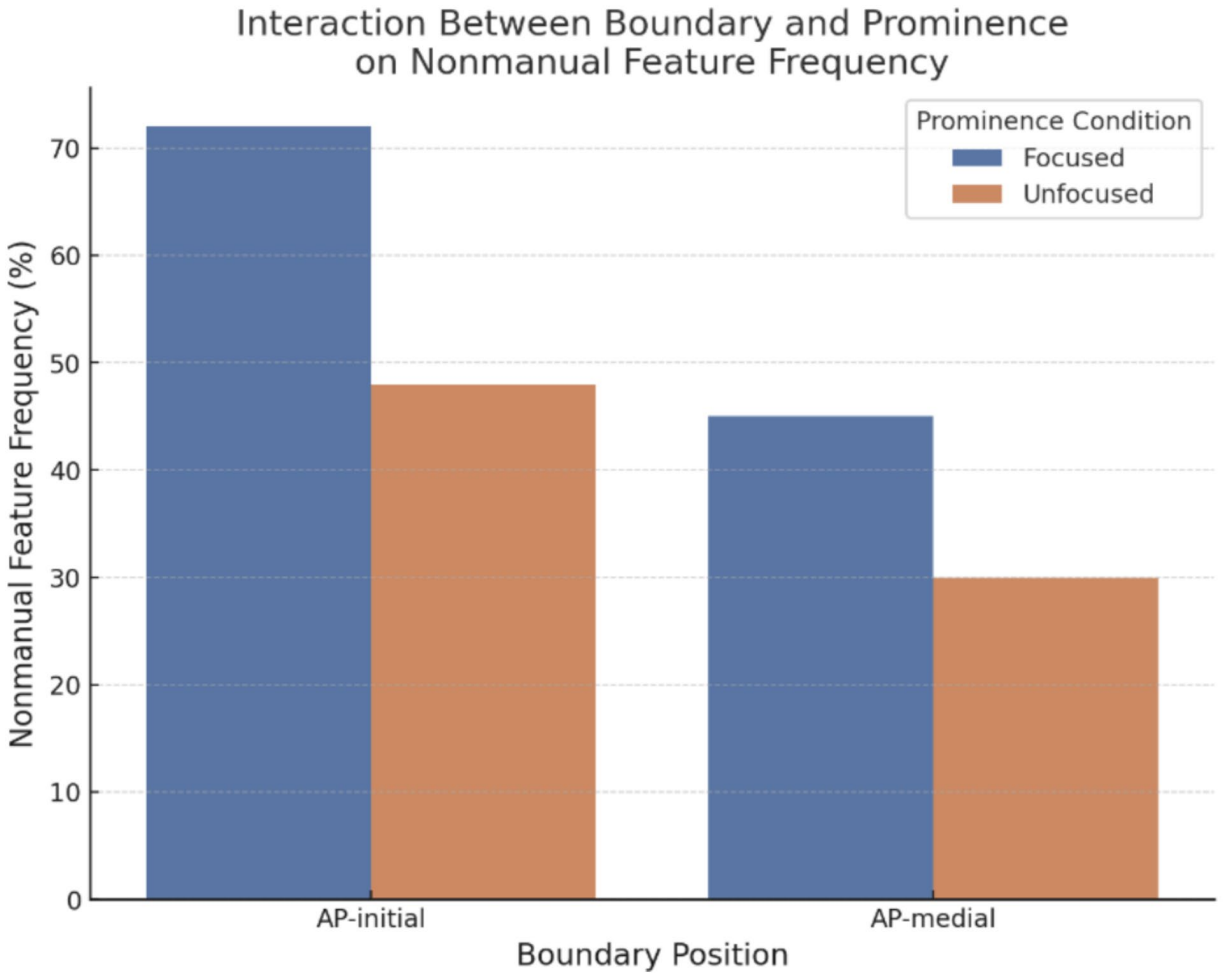
Interaction between boundary and prominence on (non)manual feature frequency.

Figure 10 presents the frequency of (non)manual features across prominence and boundary conditions. First, Table 4 presents the effects of prominence (focused vs. unfocused). Prominence showed significant and systematic effects on (non)manual features. Several nonmanual features were significantly more frequent in focused conditions, including eye contact (*β* = −1.54, SE = 0.46, *p* < 0.01), furrowed eyebrows (*β* = −1.50, SE = 0.47, *p* < 0.01), raised eyebrows (*β* = −2.43, SE = 0.57, *p* < 0.001), squinted eyes (*β* = −2.07, SE = 0.70, *p* < 0.01), wide eyes (*β* = −2.97, SE = 0.72, *p* < 0.001), head down (*β* = −1.24, SE = 0.58, *p* = 0.033), and leaning left (*β* = −1.73, SE = 0.56, *p* < 0.01). In contrast, features such as head up (*β* = 0.82, SE = 1.29, *p* > 0.05) and intense signing (*β* = 0.38, SE = 0.77, *p* > 0.05) did not differ significantly between focused and unfocused conditions. These results underscore prominence as a key driver of nonmanual feature use in KSL, particularly for eye- and eyebrow-related movements.

**Table 4:** Prominence effects on (non)manual features.

(Non)manual feature	*β*	SE	95% CI (Lower)	95% CI (Upper)	*p*-value
Eye contact	−1.54	0.46	−2.44	−0.64	0.001
Wide eyes	−2.97	0.72	−4.38	−1.56	<0.001
Squinted eyes	−2.07	0.7	−3.44	−0.70	0.003
Furrowed eyebrows	−1.5	0.47	−2.42	−0.58	0.001
Raised eyebrows	−2.43	0.57	−3.55	−1.31	<0.001
Head down	−1.24	0.58	−2.38	−0.10	0.033
Head up	0.82	1.29	−1.71	−3.35	0.525
Leaning left	−1.73	0.56	−2.83	−0.63	0.002
Intense signing	0.38	0.77	−1.13	1.89	0.622

Notes: Coefficients (β) are reported with SE, two-sided Wald 95% CIs (β ± 1.96·SE), and p-values. Reference levels:Boundary = AP-medial, Prominence = Unfocused. Thus a negative β for Prominence indicates higher frequency in Focused than in Unfocused; a positive β for Boundary indicates AP-initial > AP-medial. Minus signs are true “−”; SE/CI rounded to two decimals.

Next, the effects of boundary position (AP-initial vs. AP-medial) are summarized in Table 5. Overall, there was limited evidence supporting a significant increase in the frequency of (non)manual features in AP-initial positions compared to AP-medial positions. Moreover, most coefficients associated with AP-initial conditions were close to zero and statistically insignificant. Specifically, eye contact (*β* = 0.20, SE = 0.48, *p* > 0.05) and furrowed eyebrows (*β* = −0.10, SE = 0.48, *p* > 0.05) showed no significant differences between positions. However, squinted eyes tended to be less frequent in AP- initial positions, but this did not reach significance (*β* = −2.32, 95% CI [−4.93, 0.29], *p* = 0.081). Features such as head up (*β* = 1.93, SE = 1.40, *p* > 0.05) and intense signing (*β* = 0.72, SE = 0.89, *p* > 0.05) displayed non-significant trends toward increased frequency in AP-initial positions, suggesting weak but not robust boundary-marking tendencies.

**Table 5:** Boundary effects on (non)manual features.

(Non)manual feature	*β*	SE	95% CI (Lower)	95% CI (Upper)	*p*-value
Eye contact	0.2	0.48	−0.74	1.14	0.677
Wide eyes	0.2	0.59	−0.96	1.36	0.735
Squinted eyes	−2.32	1.33	−4.93	0.29	0.081
Furrowed eyebrows	−0.1	0.48	−1.04	0.84	0.835
Raised eyebrows	0.71	0.54	−0.35	1.77	0.189
Head down	0.16	0.61	−1.04	1.36	0.793
Head up	1.93	1.4	−0.81	4.67	0.168
Leaning left	−0.09	0.61	−1.29	1.11	0.883
Intense signing	0.72	0.89	−1.02	2.46	0.419

Notes: Coefficients (β) are reported with SE, two-sided Wald 95% CIs (β ± 1.96·SE), and p-values. Reference levels:Boundary = AP-medial, Prominence = Unfocused. Thus a negative β for Prominence indicates higher frequency in Focused than in Unfocused; a positive β for Boundary indicates AP-initial > AP-medial. Minus signs are true “−”; SE/CI rounded to two decimals.

Finally, the interaction between prominence and boundary conditions was examined (Table 6) to assess whether prominence systematically increased nonmanual feature usage specifically at AP-initial boundaries. At the feature level, all Boundary × Prominence coefficients had 95% confidence intervals that overlapped zero (Table 6), indicating no reliable interactions for individual features. By contrast, the model-level interaction term was statistically significant (*β* = 1.10, *p* < 0.05), but this average effect did not translate into consistent, feature-specific patterns. For instance, eye contact (*β* = −0.12, SE = 0.53, *p* > 0.05), furrowed eyebrows (*β* = 0.25, SE = 0.54, *p* > 0.05), and head up (*β* = −2.29, SE = 1.49, *p* > 0.05) were not significantly affected by the interaction between boundary and prominence. Thus, while prominence substantially increased the frequency of (non)manual features, this effect did not uniformly depend on boundary position, suggesting that prominence operates relatively independently from boundary cues in KSL prosody.^6^ Taken together, these results suggest that prominence robustly increases (non)manual frequency irrespective of boundary position, with no systematic amplification at AP-initial.

**Table 6:** Boundary x prominence effects on (non)manual features.

(Non)manual feature	*β*	SE	95% CI (Lower)	95% CI (Upper)	*p*-value
Eye contact	−0.12	0.53	−1.16	0.92	0.821
Wide eyes	0.57	0.85	−1.10	2.24	0.502
Squinted eyes	1.93	1.48	−0.97	4.83	0.192
Furrowed eyebrows	0.25	0.54	−0.97	1.31	0.643
Raised eyebrows	−0.41	0.7	−1.78	0.96	0.558
Head down	−0.23	0.71	−1.62	1.16	0.746
Head up	−2.29	1.49	−5.21	0.63	0.124
Leaning left	0.11	0.74	−1.34	1.56	0.882
Intense signing (manual feature)	−0.96	0.96	−2.84	0.92	0.317

Notes: Coefficients (β) are reported with SE, two-sided Wald 95% CIs (β ± 1.96·SE), and p-values. Reference levels:Boundary = AP-medial, Prominence = Unfocused. Thus a negative β for Prominence indicates higher frequency in Focused than in Unfocused; a positive β for Boundary indicates AP-initial > AP-medial. Minus signs are true “−”; SE/CI rounded to two decimals.

To support the statistical findings, we provide illustrative examples showing how (non)manual features varied across prominence and boundary conditions. In (5) how nonmanual markers are used for silly under the AP-initial focused condition is presented. In this condition, signers consistently produced a cluster of (non)manual cues associated with prominence—specifically mouthing, furrowed eyebrows, raised eyebrows, head up, and eye contact. These nonmanual signals were frequently accompanied by manual articulatory features, such as a slower signing rate and sign lengthening. Together, these observations suggest that prosodic prominence in KSL is expressed through the coordinated use of both manual and nonmanual cues, particularly when a focused element occurs at the beginning of an Accentual Phrase.

(5) AP-initial positions (Focused)

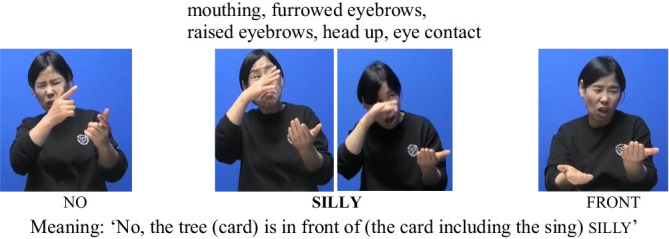


Next, in the AP-initial unfocused condition, only raised eyebrows and head lean left were observed. Notably, eye contact was not present, as seen in (6). This suggests that, in the absence of prominence, certain nonmanual cues—such as eye contact—may not be triggered, even at AP-initial positions.

(6) AP-initial positions (Unfocused)

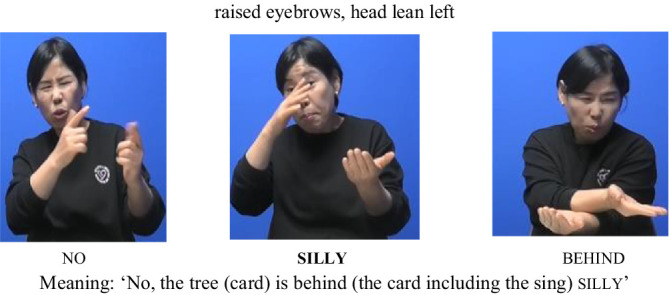


In the AP-medial focused condition, the sign silly was accompanied by raised eyebrows, eye contact, and mouthing, as seen in (7). This indicates that, even in AP-medial positions, eye contact can still emerge as a cue for prominence when the sign is in focus.

(7) AP-medial positions (Focused)

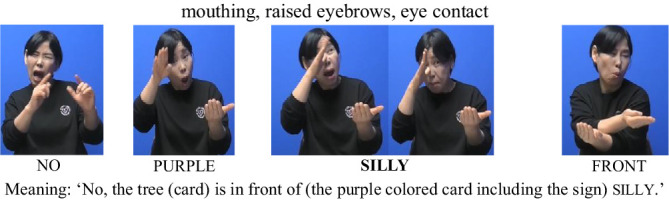


Finally, in the AP-medial unfocused condition, only mouthing was observed, with minimal use of other nonmanual feature, as seen in (8). Again, prominence-related cues such as eye contact were absent.

(8) AP-medial positions (Unfocused)

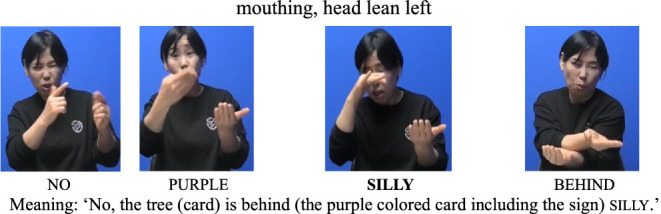


These examples are consistent with the quantitative findings, reinforcing the interpretation that prominence plays a more decisive role than boundary position in shaping the distribution of nonmanual features in KSL. Eye-related cues, in particular, were more consistently observed under focused conditions regardless of AP position, whereas postural and head movement cues appeared more frequently at AP-initial boundaries.

Taken together, these patterns suggest that prominence exerts a stronger and more systematic influence on nonmanual articulation than boundary position, with minimal interaction between the two factors.

## 4 Discussion and conclusion

Our findings clearly demonstrate that nonmanual prosody in KSL is not a random or stylistic choice, but rather a systematic and functionally organized component of the language’s prosodic system. Statistical analyses revealed that both prominence (focused vs. unfocused) and boundary position (AP-initial vs. AP-medial) significantly influenced the frequency and type of nonmanual features observed. Specifically, focused signs consistently exhibited a higher frequency of nonmanual cues—such as eye contact, furrowed eyebrows, raised eyebrows, wide eyes, head down, and leaning left—compared to unfocused signs. These results confirm that nonmanual prosody in KSL plays a critical role in prominence marking, particularly through facial and head articulations.

A feature-specific analysis further revealed that while most nonmanual cues were closely associated with prominence, squinted eyes functioned as a boundary cue, appearing predominantly in AP-initial positions and rarely in AP-medial focused conditions. This fine-grained division of prosodic functions highlights the systematic nature of nonmanual prosody in KSL and underscores the language-specific role of facial and head movements. Unlike previous findings in ISL, where eyebrow movements primarily marked boundaries (Dachkovsky and Sandler, 2009), KSL signers predominantly used eyebrow and eye movements as prominence markers. This suggests that prosodic cues are subject to cross-linguistic and cross-modal variation.

Our findings align with Crasborn and van der Kooij (2013) observations in NGT, where prominence marking commonly involves the co-occurrence of multiple nonmanual cues rather than a single isolated signal. KSL signers similarly used coordinated facial and head articulations, supporting the view that prominence in sign languages is typically realized through multimodal enhancement.

Importantly, our study contributes to the ongoing theoretical debate on whether focus necessarily triggers prosodic restructuring. In spoken Korean, Jun (2006) argued that focus induces the formation of a new AP boundary, whereas Cho (2022) demonstrated that prominence can also be realized without prosodic restructuring. Extending this debate to KSL, our results suggest that in KSL, prominence is primarily marked through nonmanual cues independent of prosodic boundary formation, aligning with Cho (2022) view. This finding indicates that prominence and boundary marking in KSL are functionally more differentiated, and that prosodic restructuring is not a prerequisite for marking prominence.

Taken together, these findings provide empirical evidence that prosodic strategies in KSL are modality-specific, with a unique emphasis on facial and head movements rather than on spatial or temporal adjustments commonly reported in other sign languages. The systematic use of nonmanual cues in KSL reflects both universal prosodic strategies and language-specific adaptations shaped by the visual-manual modality and the cultural-linguistic context of the Korean Deaf community.

Future studies should further explore larger prosodic units (e.g., IP boundaries) and more spontaneous signing to assess the generalizability of the current findings. Perceptual studies could also investigate how KSL users process nonmanual prosodic cues, providing deeper insights into cue salience and cognitive processing. Additionally, cross-linguistic studies across sign languages (e.g., ASL, JSL, and BSL) and research on the acquisition of nonmanual prosody in KSL learners will be valuable for understanding both universal and language-specific aspects of prosody.

In sum, this study provides the first systematic phonetic-prosodic analysis of prominence and boundary marking in KSL. By demonstrating that prominence can be realized independently of prosodic restructuring, we contribute to a growing body of work that emphasizes the flexibility of prosodic systems across modalities. Our findings position KSL within broader typological and theoretical frameworks of prosody, offering new insights into the cross-modal diversity of prosodic organization.

## Data Availability

The datasets, analysis scripts and anonymized annotations presented in this study can be found in online repositories. This data can be found here: https://osf.io/9duze/?view_only=5c452fd5002a480588b85148ff555019.

## Footnotes

1 While these participants share similar early exposure to KSL, we avoid the term “native signer*”* in line with recent discussions in the field (Bisnath, 2024; Hou and Namboodiripad, 2025), which highlight the diverse pathways of sign language acquisition and call for more precise descriptions of signing backgrounds.

2 We acknowledge that the presence and behavior of the interviewer may have influenced participants’ use of nonmanual cues during the interaction. However, note that was a close hearing family member of a deaf signer and lives in daily contact with the Deaf community. Although not a deaf signer, she is fluent in KSL due to regular interaction with native signers, including her spouse who is also a signer. Future research could systematically control and analyze for interviewer effects to better isolate participant-driven prosodic patterns.

3 In KSL, chicken and silly share the same handshape, movement, and palm orientation, but differ in location: chicken is articulated on the forehead, while silly is signed on the nose. See and find differ only in movement, while sharing the same handshape, orientation, and location, making them a minimal pair based on movement. The signs practice and non-deaf differ in palm orientation, while maintaining the same handshape, movement, and location, constituting a minimal pair based on orientation. Finally, pretty and difficult form a minimal pair based on handshape, as all other parameters remain identical.

4 Available at: https://osf.io/9duze/?view_only=5c452fd5002a480588b85148ff555019.

5 Although the stimulus sentences were designed to elicit prosodic variation, prosodic segmentation was determined solely by the prosodic patterns that naturally emerged in the signers’ spontaneous signing, rather than by syntactic structure. To our knowledge, this is the first study to systematically investigate prosodic boundaries and units in KSL using a phonetic-prosodic approach. Prior research on KSL prosody is extremely limited. Some studies have informally distinguished smaller prosodic units such as the Accentual Phrase (AP) below the Intonational Phrase (IP), and the National Institute of Korean Language (2021) briefly notes that phrase boundaries in KSL may be indicated by pauses and final sign lengthening, particularly preceding IP-initial positions. In KSL, it has been reported that pauses and manual cues such as sign lengthening often occur before IP-initial positions. However, little is known about how nonmanual cues—such as head movements, facial expressions, and eye gaze—are distributed at prosodic boundaries, especially between the preceding phrase and the AP-initial position. The current study addresses this gap by independently identifying prosodic units based on observable patterns in natural KSL signing and by systematically analyzing the role of nonmanual cues in boundary marking at the AP level. For the prosodic unit of analysis, we adopted the AP, following empirical observations from the Chosun University Korean Sign Language Corpus. (Non)manual cues were identified based on observable visual cues and were independently validated by a professional KSL interpreter, the second author (a sign language specialist), and native KSL signers.

6 Data analysis was conducted by the KSL interpreter. To ensure data reliability, all annotations and analyses were cross-checked by the second author, director of the sign language linguistics research lab and a certified KSL researcher (IRB: 2-1041055-AB-N-01-2024-24).
